# Multi-Flap Microsurgical Autologous Breast Reconstruction

**DOI:** 10.3390/jcm13175324

**Published:** 2024-09-09

**Authors:** Thomas N. Steele, Sumeet S. Teotia, Nicholas T. Haddock

**Affiliations:** Department of Plastic Surgery, University of Texas Southwestern, Dallas, TX 75390, USA; thomassteelemd@gmail.com (T.N.S.); sumeet.teotia@utsouthwestern.edu (S.S.T.)

**Keywords:** breast reconstruction, microsurgery, autologous, free flap, stacked, conjoined, double pedicle, DIEP, PAP, LAP

## Abstract

Microsurgical autologous breast reconstruction (MABR) remains the gold standard technique of breast reconstruction, providing a durable, natural, and aesthetically pleasing result. However, some patients may not be candidates for a traditional deep inferior epigastric perforator (DIEP) flap, either due to abdominal tissue paucity, the need for higher-volume reconstruction, or prior surgical procedures. In these patients, alternative flaps must be considered to achieve the optimal result. Such configurations include the conjoined (or double pedicle) DIEP flap, and alternative flaps such as the lumbar artery perforator (LAP) and profunda artery perforator (PAP) flaps, which can be combined in a stacked fashion. By combining multiple flaps in a conjoined or stacked fashion, breast reconstruction can be optimized to fulfill the three critical components of breast reconstruction in restoring the skin envelope, breast footprint, and conus shape. When harvesting multiple flaps, the surgical sequence of events must be meticulously planned to ensure an efficient and successful operation. Preoperative imaging can aid the surgeon in identifying the ideal perforator, assess for side branches for possible intra-flap anastomoses, expedite the operative time, and decrease intraoperative complications. Reconstructive surgeons should be familiar with the variety of configurations with conjoined and/or stacked flaps to address patient-specific reconstructive needs.

## 1. Introduction

The evolving landscape of breast reconstruction has been marked by significant advancements in autologous free tissue transfer [1]. Historically, microsurgical autologous breast reconstruction (MABR) using the abdominal donor site has been the gold standard, largely due to its favorable outcomes in terms of donor site morbidity, complication rates, and patient satisfaction [2,3,4]. However, challenges arise in achieving an aesthetically satisfactory reconstruction, particularly for patients with limited abdominal tissue or those undergoing delayed reconstruction post-radiotherapy. These situations often necessitate alternative approaches to adequately address the restoration of the breast’s “footprint”, “conus”, and “skin envelope”—essential elements in replicating a natural breast appearance [5].

The advent of novel perforator flap techniques has provided new avenues for bilateral breast reconstruction, particularly for patients with minimal donor abdominal tissue who wish to avoid prosthetic implants [6,7,8,9,10,11]. These techniques not only offer additional skin to create a more natural breast envelope with appropriate ptosis but also provide sufficient volume for creating body-appropriate breast mounds, thereby reducing the need for unpredictable fat grafting procedures. The practice of using multiple flaps, including stacked configurations of deep inferior epigastric perforator (DIEP) or transverse rectus abdominis musculocutaneous (TRAM) flaps, has been further refined with the development of alternative flaps, particularly the profunda artery perforator (PAP) flap and the lumbar artery perforator (LAP) flap [12]. This evolution reflects the growing demand for alternative autologous reconstruction options, particularly among patients with contralateral or bilateral prophylactic mastectomies.

In addressing the complex requirements of breast reconstruction, especially in cases involving significant skin deficits or the need for additional tissue in the upper pole of the breast, the strategy of using stacked or conjoined flaps has emerged as a highly effective approach [13,14,15,16,17,18,19,20]. This method not only provides the necessary skin envelope and volume but also offers a tailored solution based on individual patient needs. While alternative methods like fat grafting, pedicled flaps, or the use of implants can be successful in specific scenarios, they often carry higher risks of unpredictability and potential morbidity. The use of multiple free flaps in a stacked or conjoined fashion has shown considerable promise in achieving aesthetically satisfactory results, both for unilateral and bilateral reconstructions.

## 2. Clinical Considerations

### 2.1. Definitions

The term “stacked flap” has been used to describe a variety of procedures, typically referring to the use of more than one flap to reconstruct a single breast. In the classical sense, “stacked” means placing one object on top of another. But regarding autologous breast reconstruction, placing one flap on top of another is rarely the case. The following definitions are used in the authors’ practice to simplify this concept:

Conjoined Flap: Double-pedicle flaps from the same donor site (e.g., a double-pedicle DIEP flap or a PAP flap with additional medial circumflex femoral pedicle).

Stacked Flap: Two separate flaps with separate pedicles from separate donor sites (e.g., two separate PAP flaps for a single breast, or a DIEP and PAP flap used for unilateral breast reconstruction).

At the authors’ institution, 28% (818 flaps) of all MABR is performed in a stacked/conjoined fashion. In a previous review of the authors’ clinical experience, there was no detectable increase in donor site wound complications [18].

### 2.2. Background

Three critical considerations of breast reconstruction include the breast footprint, the skin envelope, and the conus shape [21]. Of these three factors, the skin envelope and footprint are of paramount importance at the index reconstruction. Following a skin-sparing mastectomy technique, a single breast can often be reconstructed with a single flap. But in many instances, when one considers the impact of radiation, contralateral breast volume and shape, and patient preference, more tissue is often required than can be provided with a single flap. When multiple flaps in either stacked or conjoined fashion are used, all core aspects of breast reconstruction can be achieved, even in difficult cases.

In cases of significant skin deficit, as can occur in delayed breast reconstruction or in the irradiated breast, a significant portion of skin is often required, usually at the inferior pole of the breast. While a single flap can provide enough skin for this, the breast footprint is often compromised, particularly at the upper pole of the breast. Some surgeons propose autologous fat grafting to augment this region, but resorption rates are high in breasts lacking an adequate scaffold for fat revascularization [22]. Furthermore, lipoaspirate donor sites are not without complications, including ecchymoses, pain, and hematoma as the most commonly reported downsides [23].

The use of multiple flaps either in a conjoined or stacked fashion allows for a tailored and patient-centric approach and provides all core components of total breast reconstruction. As many surgeons can attest, having excess tissue allows for a more straightforward revision in the form of a lift or reduction.

### 2.3. Patient Indications

As discussed previously, the ideal reconstructed breast has a stable foundation on the chest wall, appropriate volume in all four breast quadrants to cause projection from the chest wall, and an appropriate skin envelope to allow for natural ptosis [5]. The patients requiring a stacked or conjoined flap for ideal breast reconstruction fall into three categories: (1) the delayed patient with contracted skin and a moderate- to large-sized contralateral breast; (2) the irradiated patient with inadequate skin and inhospitable scaffold for autologous fat grafting; and (3) the thin patient (BMI < 25) in whom a single hemi-abdominal donor site will not provide enough volume for an adequate reconstruction. For these patients, multiple flaps should be considered at the primary reconstruction.

## 3. Preoperative Planning

Universally, the most common MABR donor site is the abdomen in the form of a DIEP flap. In the authors’ practice, alternative donor sites almost exclusively come from the thigh (PAP) and flank (LAP) [24,25,26]. These three flaps make up nearly 100% of our MABR practice. Preoperative computed tomography angiogram (CTA) is essential for evaluating vascular anatomy and donor site volume and incorporating patient preference into decision-making [27].

For reconstructions requiring multiple flaps, two vascular recipient sources are necessary, typically utilizing the cranial and caudal internal mammary vessels. However, alternative techniques such as intra-flap pedicle extensions may be considered, especially in cases of delayed reconstruction post-radiation where the caudal internal mammary vein may be unsuitable [16,28]. In these cases, the flap is perfused through antegrade internal mammary flow to one of the flaps, and the second flap is anastomosed to an extension of the first flap’s pedicle, or a side branch of adequate caliber [29]. Alternative recipient vessels may include the lateral thoracic artery, thoracoacromial artery, or branch to serratus, but these sites may cause unaesthetic lateralization of the breast mound.

Preoperative imaging plays a crucial role in identifying potential alternative recipient sites within the flap, and identification of a side branch of adequate caliber can guide quick intraoperative decision-making. Intraoperative verification of vessel flow, via a “spurt test” or simply a visual examination of blood flow from the cut end of the artery, should always be performed prior to anastomosis. Effective communication among the surgical team is vital for coordinating recipient vessel selection and overall planning.

## 4. Operative Technique

In describing the possible configurations of multi-flap breast reconstruction, it is outside the scope of this review to intimately detail every possibility of configuration. Rather, the authors will summarize the preferred practice at our institution regarding the combination of stacked and conjoined flaps utilized on a routine basis.

### 4.1. Conjoined Deep Inferior Epigastric Artery Perforator Flap

The conjoined DIEP flap is the most common option in the armamentarium of multi-flap breast reconstruction. In this technique, two hemi-abdominal DIEP flaps are raised without separating the flap midline. As the DIEP flap has risen in popularity in teaching institutions across the country, most reconstructive surgeons are trained in this technique. The dissection of the deep inferior epigastric perforators and main pedicle is similar to a unilateral DIEP, but there are additional considerations given the added complexity [30].

The first is the dissection technique. As the midline remains attached, dissection proceeds from lateral and inferior to medial and superior until adequate perforator(s) are identified. The superior flap border is typically left attached as an additional security measure against stretching or twisting of the pedicle, and the inferior flap border is stapled to the abdominal wall to provide adequate exposure to the main pedicle.

Secondly, a key decision point is the location of the second anastomosis. The first flap is typically inset into the antegrade (cranial) internal mammary vessels. The second flap can either be anastomosed to the caudal internal mammary vessels, or to a cranial extension or side branch of the first flap’s main pedicle. The primary benefit of intra-flap anastomoses is the freedom of flap inset it provides, while the primary drawback is that in the event of vascular compromise, both flaps may be lost. The individual needs of each patient dictate the decision-making process [31].

**Figure 1:**
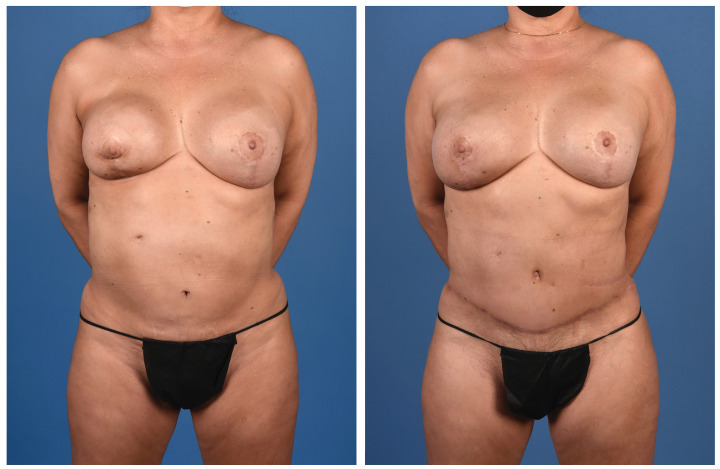
A 59-year-old woman with a history of right breast cancer treated with mastectomy and radiation. She underwent implant reconstruction at an outside hospital and presented for autologous conversion secondary to capsular contracture. She underwent a right double-pedicle conjoined DIEP flap with a left symmetry procedure.

Ultimately, the conjoined DIEP flap is the “workhorse” for unilateral breast reconstruction requiring multiple flaps. It can provide all three aspects of breast reconstruction by offering the most skin envelope of any of the flaps, adequate volume for establishing the breast footprint, and shaping of the conus. The inset can be horizontal, vertical, or oblique depending on the reconstructive needs. The horizontal and oblique inset provides the most skin, while the vertical inset allows for folding of the flap on itself to augment projection and increase upper pole fullness (Figure 1).

### 4.2. Stacked Profunda Artery Perforator Flaps

**Figure 2:**
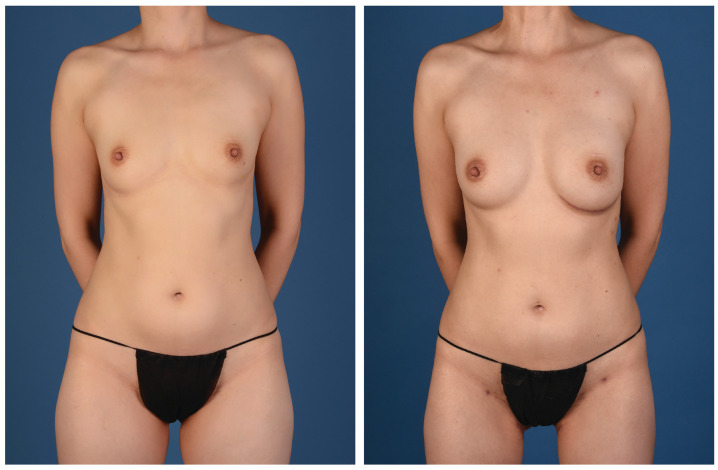
A 45-year-old woman with left breast cancer treated with delayed immediate stacked PAP flaps and symmetry fat grafting to the right breast (anterior view).

**Figure 3:**
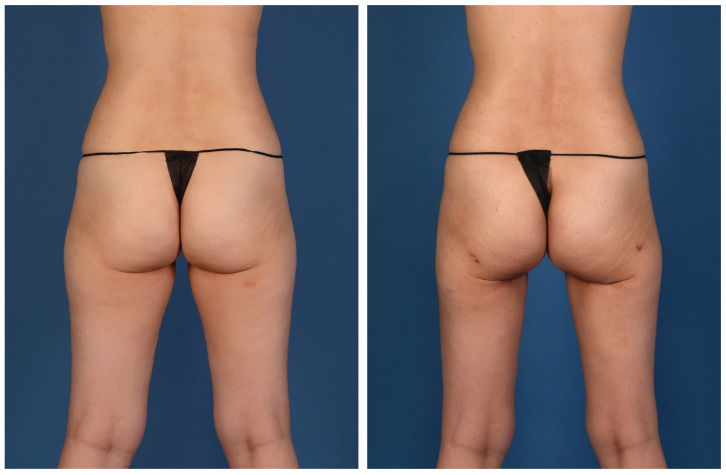
A 45-year-old woman with left breast cancer treated with delayed immediate stacked PAP flaps and symmetry fat grafting to the right breast (posterior view).

**Figure 4:**
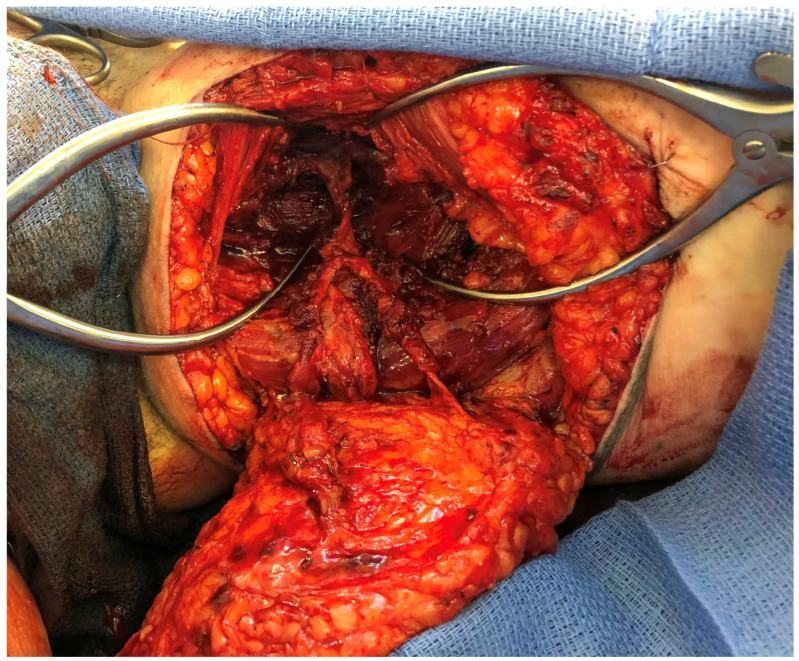
Intraoperative photo of medial femoral circumflex perforator as part of a conjoined double-pedicle inner posterior thigh flap (PAP flap).

In the low BMI patient without adequate abdominal donor tissue, or in patients with a history of abdominal surgery, the stacked PAP flap is a reliable option (Figure 2 and Figure 3). The technique for harvesting the PAP flap has been previously described by the authors [25,32]. One nuance is the careful dissection of the pedicle, during which the surgeon should examine for the presence of side branches to allow for intra-flap anastomoses. The dissection should proceed to the pedicle origin, not only for the maximum length of the main pedicle but for the fact that these side branches, if present, are only of adequate caliber close to the pedicle origin. In rare cases, the medial femoral circumflex femoral vessels can be harvested as a dual pedicle, or “conjoined” flap, but this rarely augments the skin or tissue donor site in any meaningful way (Figure 4). In our experience, this technique is used only when the main PAP vessels are diminutive and the MFC perforator is dominant at the anterior aspect of the flap.

### 4.3. Stacked Lumbar Artery Perforator Flaps

The LAP flap was first described as a free perforator flap for breast reconstruction by de Weerd in 2003 [11]. Since then, it has rapidly ascended in the authors’ practice as equivalent to the PAP flap, depending on patient donor site characteristics [26]. The stacked LAP is not common, but in the low BMI patient requiring higher-volume breast reconstruction and an adequate donor site in the “love handle” region, this is a good option. The primary drawback is the short pedicle (3–4 cm), requiring the use of an additional composite graft (artery and vein) [33]. This is harvested from the DIEA/V system most frequently, and rarely from the descending branch of the lateral circumflex femoral (LCF) system. These composite grafts can often have side branches, allowing for two flaps to be connected to one graft and subsequently one recipient site on the chest. More commonly, two grafts are harvested and anastomosed to each flap, and a cranial–caudal internal mammary vessel configuration is used. During the multiple position changes, it is imperative to communicate with team members to allow efficient and effective progression through the planned operation.

### 4.4. Stacked DIEP and PAP Flaps

**Figure 5:**
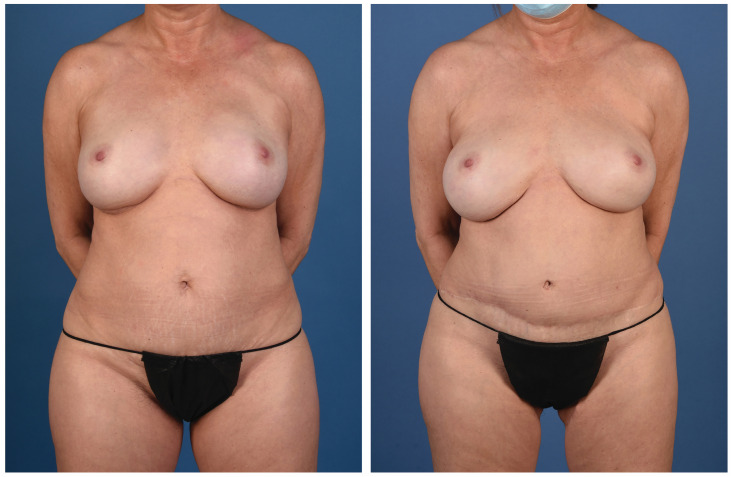
A 58-year-old female with a history of breast cancer treated with bilateral mastectomy and implant reconstruction at an outside hospital. She presented for autologous conversion and underwent bilateral stacked DIEP and PAP flaps (anterior view).

**Figure 6:**
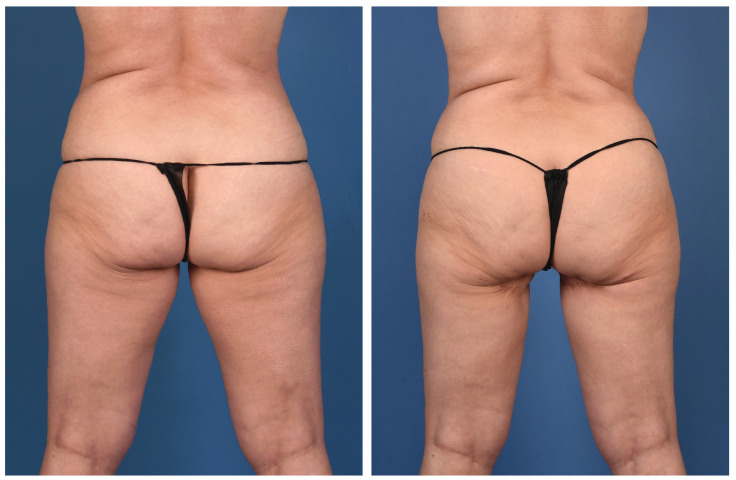
A 58-year-old female with a history of breast cancer treated with bilateral mastectomy and implant reconstruction at an outside hospital. She presented for autologous conversion and underwent bilateral stacked DIEP and PAP flaps (posterior view).

The first described four-flap breast reconstruction was the bilateral stacked DIEP and PAP flap [15,16,17]. The primary benefits of this technique are the simultaneous execution at the chest, abdomen, and thigh surgical sites, and that no position change is required. Regarding the inset of the flaps, the natural curve of the PAP flap closely matches the inferior pole of the breast, and the tapered upper pole matches that of the DIEP flap. If both flaps and pedicles are of equal caliber, this is the preferred orientation. However, more important in the authors’ opinion is identifying the larger or more dominant flap, which is then anastomosed to the cranial system for increased reliability. The DIEP pedicle commonly has multiple side branches or a cranial extension that can be used for “parasitic” perfusion via intra-flap anastomoses, with the main DIEP vessels connected to the dominant cranial internal mammary system. While the additional surgical sites increase the postoperative complication risk, the use of two flaps allows for less volume/skin requirement, thereby decreasing tension at the donor site closure (Figure 5 and Figure 6).

### 4.5. Stacked DIEP and LAP Flaps

When considering the truncal contour of a patient, there are perhaps no greater aesthetic donor sites than the stacked DIEP and LAP flaps [34]. This combination not only provides significant tissue volume and skin envelope for reconstructing the aesthetic breast mound, but it is also effectively a belt lipectomy, with lower trunk aesthetic results like a circumferential body lift. While the aesthetic benefits are clear, there are significant drawbacks, mainly regarding the vascular pedicle of the LAP and sequencing of the operation.

As discussed earlier, all LAP flaps require a composite vessel graft to extend the pedicle length. But in this case, the preferred donor site of the DIEP is not available. The side branches or cranial extension of the DIEP pedicle are available as intra-flap anastomosis to the LAP, but this can shorten the working pedicle of both flaps and make the inset difficult. The preferred second choice is the descending branch of the LCF vessels, but this has the downside of adding an additional scar to the anterolateral thigh.

**Figure 7:**
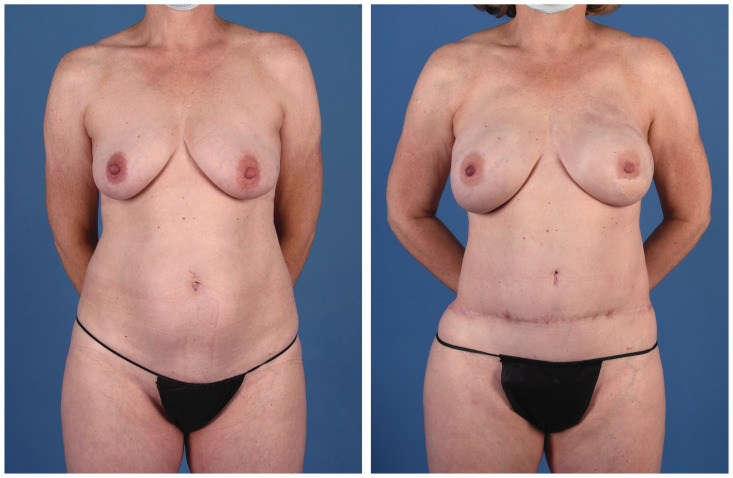
A 56-year-old woman with a genetic predisposition to breast cancer treated with prophylactic nipple-sparing mastectomy and tissue expanders. This was followed by autologous reconstruction with bilateral stacked DIEP and LAP flaps (anterior view).

**Figure 8:**
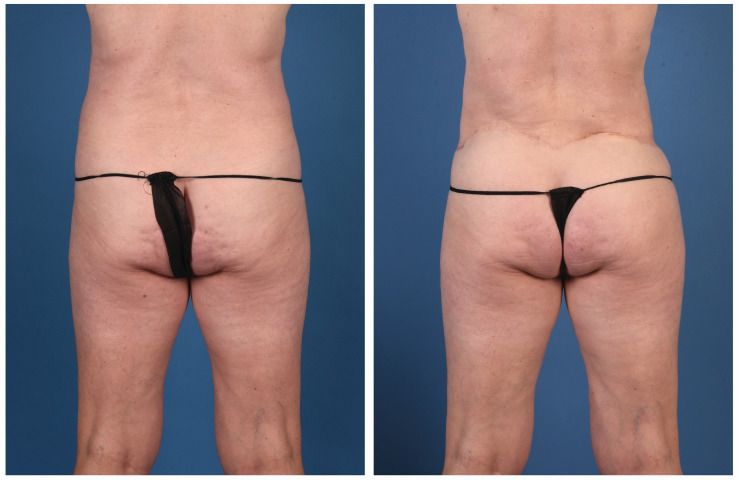
A 56-year-old woman with a genetic predisposition to breast cancer treated with prophylactic nipple-sparing mastectomy and tissue expanders. This was followed by autologous reconstruction with bilateral stacked DIEP and LAP flaps (posterior view).

Furthermore, the timing of the surgical sequence must be considered. If the DIEP flaps are harvested and anastomosed to the mammary vessels first, the surgeon must accept the repositioning of the patient in the prone position to harvest the LAP flaps immediately after multiple fresh anastomoses. Alternatively, the DIEP flap harvest can be delayed until after the LAP flaps are harvested, the back is closed, and the flaps are re-perfused on the chest. This sequence adds to the overall operative duration, and the DIEP flaps are perfused on a non-dominant superior supply during the multiple position changes. Regardless of the preferred sequence of events, efficiency and effective communication remain paramount to a successful operation (Figure 7 and Figure 8).

### 4.6. Stacked PAP and LAP Flaps

**Figure 9:**
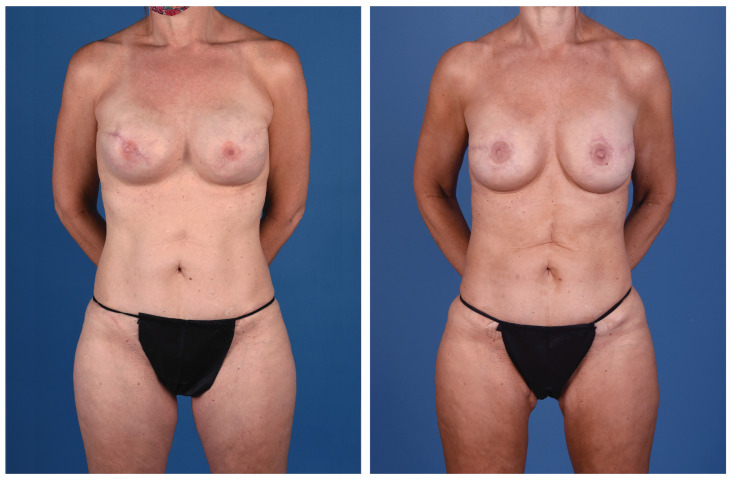
A 56-year-old woman with a genetic predisposition to breast cancer treated with prophylactic nipple-sparing mastectomy and implant-based reconstruction at an outside hospital. She presented for autologous conversion and underwent bilateral stacked LAP and PAP flaps (anterior view).

**Figure 10:**
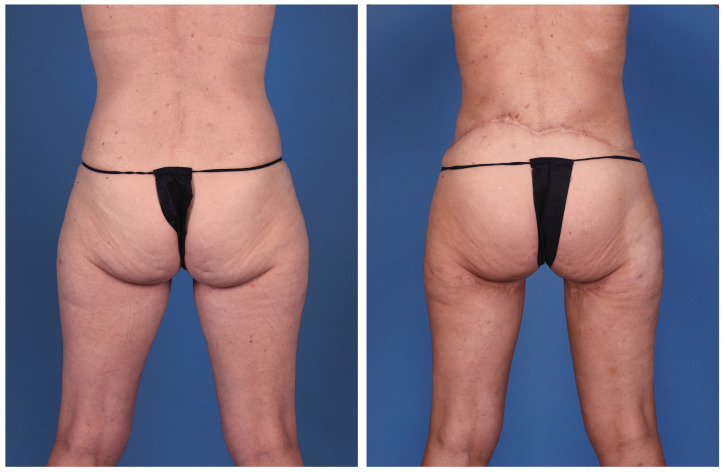
A 56-year-old woman with a genetic predisposition to breast cancer treated with prophylactic nipple-sparing mastectomy and implant-based reconstruction at an outside hospital. She presented for autologous conversion and underwent bilateral stacked LAP and PAP flaps (posterior view).

**Figure 11:**
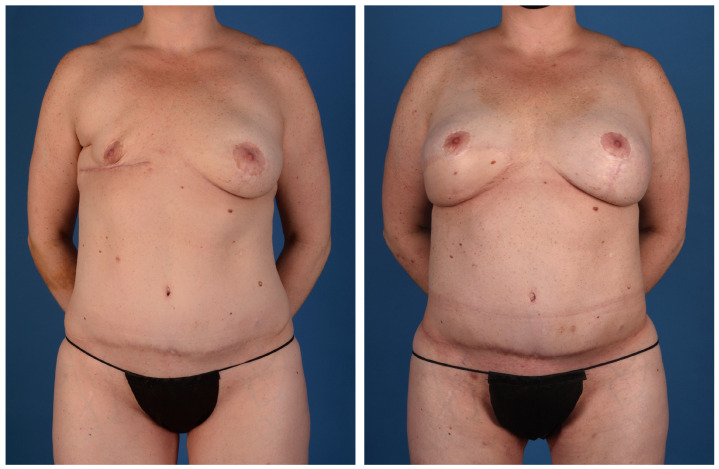
A 48-year-old woman with a history of breast cancer treated with bilateral nipple-sparing mastectomy and tissue expander-based reconstruction at an outside hospital. Her right breast reconstruction was complicated by mycobacterium infection, and ultimately, the expander was lost. She had undergone a previous abdominoplasty. She presented for autologous reconstruction and underwent bilateral stacked LAP and PAP flaps (anterior view).

**Figure 12:**
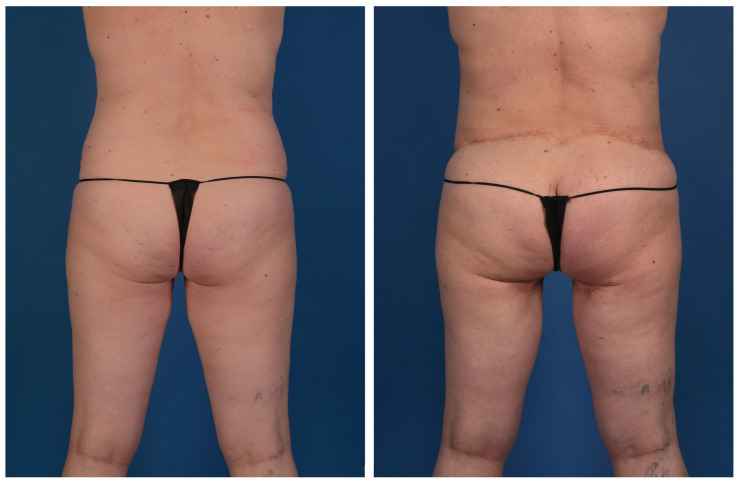
A 48-year-old woman with a history of breast cancer treated with bilateral nipple-sparing mastectomy and tissue expander-based reconstruction at an outside hospital. Her right breast reconstruction was complicated by mycobacterium infection, and ultimately, the expander was lost. She had undergone a previous abdominoplasty. She presented for autologous reconstruction and underwent bilateral stacked LAP and PAP flaps (posterior view).

The stacked PAP and LAP flap is rarely used in our practice, reserved for patients who have a paucity of donor site tissue at a single site and who have had a prior abdominoplasty. Again, the surgical sequence of events is the main consideration. The PAP flaps can be harvested first and anastomosed to the chest or delayed until after the LAP flaps are harvested. Proceeding with the harvest of the PAP flaps first allows for an easier donor site closure when converted to the prone position. Otherwise, the same factors as discussed previously should be considered (Figure 9, Figure 10, Figure 11 and Figure 12).

## 5. Postoperative Considerations

The execution of stacked flap breast reconstruction is one of the most, if not the most, technically demanding methods of breast restoration today. There are many nuances to each step of the operation, and as surgical complexity increases, the risk of complications rises. A recent retrospective comparison of stacked flaps to single flaps at our institution revealed an increased rate of deep venous thrombosis in stacked flap patients [18]. It is routine now for patients to start a two-week course of rivaroxaban on postoperative day 1 to mitigate this risk.

In the same study, an increased rate of return to the operating room was present, but this was not associated with increased rates of flap loss. Considering the inset of stacked flaps, one flap is often buried in the superior aspect of the reconstructed breast, and implantable Doppler monitoring may yield “false alarms” at a slightly higher rate when there is no clinical exam upon which to rely. Subtle changes in the audible Doppler exam may result in a return to the operating room and ultimately a negative exploration simply to confirm viability.

Our institution has adopted the now widespread practice of the Enhanced Recovery After Surgery (ERAS) pathway [35]. Preoperative factors include the administration of celecoxib and acetaminophen as well as allowing patients to drink 12 ounces of an electrolyte-rich carbohydrate beverage. Intraoperative factors include the use of regional field blocks using local analgesics such as liposomal bupivacaine or Marcaine. Administration of intravenous steroids and Ondansetron as well as a scopolamine patch are useful to prevent postoperative nausea. Postoperative milestones include early ambulation and early oral intake as well as administration of non-narcotic analgesics as needed. Our practice has witnessed remarkably improved patient-reported outcomes with the use of intraoperative transversus abdominis plane (TAP) blocks using liposomal bupivacaine [36].

## 6. Results

At the authors’ institution, nearly 3000 flaps have been performed for MABR over a 12-year period (2012–2023). Approximately 28% of flaps are performed in a stacked/conjoined fashion. For patients undergoing stacked/conjoined flap breast reconstruction, the average age is 50.4 years old (±9.2), BMI is 27.0 (±3.7), and the majority are white/Caucasian ethnicity (76.7%). Hypertension is present in 11.8% of patients, and diabetes is present in 1.4%. Radiation was performed in 51.8% of patients undergoing stacked/conjoined flap reconstruction.

**Figure 13:**
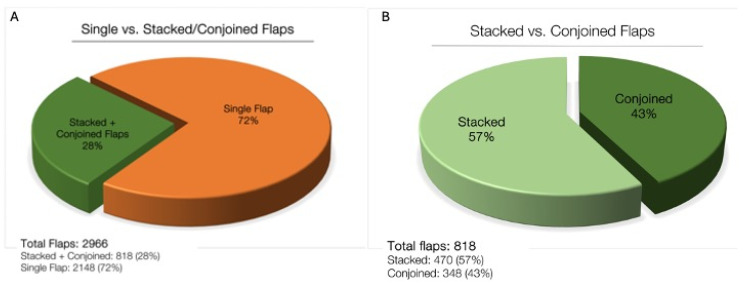
(**A**) A review of all MABR flaps performed at a single institution over 12 years (2012–2023). (**B**) Number of stacked versus conjoined flaps.

**Figure 14:**
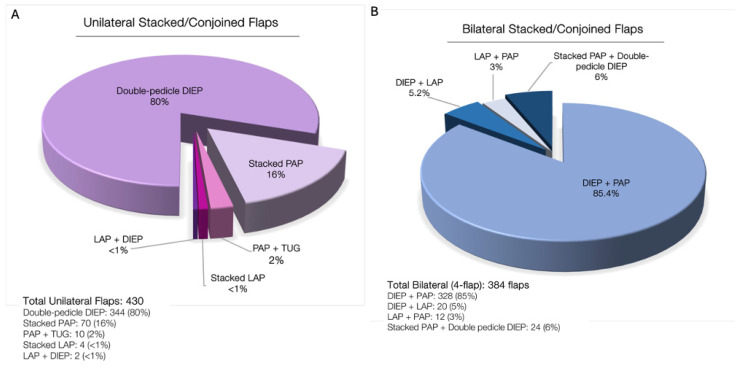
(**A**) Unilateral stacked/conjoined flaps: 80% of flaps are double-pedicle DIEP flaps (conjoined DIEP), 16% are stacked PAP flaps, 2% are PAP and TUG, <1% are stacked LAP flaps, and <1% are stacked LAP and DIEP flaps. (**B**) Most bilateral MABR cases are stacked DIEP and PAP flaps (85.4%), with 6% being stacked PAP and conjoined DIEP, 5% being DIEP and LAP, and 3% being stacked LAP and PAP.

Of 818 stacked/conjoined flaps, 57% are stacked while 43% are conjoined (Figure 13). When divided into unilateral versus bilateral cases, most unilateral cases (80%) are performed using a double-pedicle DIEP. In bilateral cases, the combination of DIEP + PAP is used in over 85% of cases (Figure 14).

Complication rates were categorized based on major criteria, which is defined as a complication requiring a return to the operating room, or minor criteria, defined as a complication that can be managed in a clinic. Overall complication rates (minor and major) are slightly higher in stacked/conjoined flap reconstruction (33.4%) compared to single flap (27.7%), largely due to breast wound dehiscence (*p* < 0.05). There were no statistical differences in age, comorbidities, neoadjuvant chemotherapy, or flap failure between stacked/conjoined flap and single-flap reconstructions.

## 7. Discussion

Stacked and conjoined flaps represent a pivotal development in autologous breast reconstruction, particularly for patients facing significant discrepancies between donor tissue availability and the desired breast volume. This technique not only provides sufficient breast reconstruction volume in a single surgical procedure without the need for synthetic implants but also demonstrates a lower incidence of complications, such as fat necrosis, when compared to other methods like extended hemi-abdominal flaps, which extend across the midline.

The art of reconstructive breast surgery lies in the proper selection of cases in which a conjoined or stacked flap should be implemented. By providing a general overview of several different combinations of flap type, while also providing a graphical breakdown of flap combinations most utilized in our institution, the authors hope to simplify the nuanced topic of multi-flap breast reconstruction for the reconstructive surgeon.

## Data Availability

No new data were created or analyzed in this study. Data sharing is not applicable.

## References

[1] Healy C., Allen R.J. (2014). The Evolution of Perforator Flap Breast Reconstruction: Twenty Years after the First DIEP Flap. J. Reconstr. Microsurg..

[2] Chang E.I. (2021). Latest Advancements in Autologous Breast Reconstruction. Plast. Reconstr. Surg..

[3] Khajuria A., Prokopenko M., Greenfield M., Smith O., Pusic A.L., Mosahebi A. (2019). A Meta-Analysis of Clinical, Patient-Reported Outcomes and Cost of DIEP versus Implant-Based Breast Reconstruction. Plast. Reconstr. Surg. Glob. Open.

[4] Kroll S.S., Baldwin B. (1992). A Comparison of Outcomes Using Three Different Methods of Breast Reconstruction. Plast. Reconstr. Surg..

[5] Blondeel P.N., Hijjawi J., Depypere H., Roche N., Van Landuyt K. (2009). Shaping the Breast in Aesthetic and Reconstructive Breast Surgery: An Easy Three-Step Principle. Plast. Reconstr. Surg..

[6] Allen R.J., Treece P. (1994). Deep Inferior Epigastric Perforator Flap for Breast Reconstruction. Ann. Plast. Surg..

[7] Allen R.J., Haddock N.T., Ahn C.Y., Sadeghi A. (2012). Breast Reconstruction with the Profunda Artery Perforator Flap. Plast. Reconstr. Surg..

[8] Elliott L.F., Beegle P.H., Hartrampf C.R. (1990). The Lateral Transverse Thigh Free Flap: An Alternative for Autogenous-Tissue Breast Reconstruction. Plast. Reconstr. Surg..

[9] Tuinder S.M.H., Beugels J., Lataster A., De Haan M.W., Piatkowski A., Saint-Cyr M., Van Der Hulst R.R.W.J., Allen R.J. (2018). The Lateral Thigh Perforator Flap for Autologous Breast Reconstruction: A Prospective Analysis of 138 Flaps. Plast. Reconstr. Surg..

[10] Allen R.J., Tucker C. (1995). Superior Gluteal Artery Perforator Free Flap for Breast Reconstruction. Plast. Reconstr. Surg..

[11] de Weerd L., Elevenes O.P., Strandenes E., Weum S. (2003). Autologous Breast Reconstruction with a Free Lumbar Artery Perforator Flap. Br. J. Plast. Surg..

[12] Haddock N.T., Teotia S.S. (2023). Modern Approaches to Alternative Flap-Based Breast Reconstruction: Stacked Flaps. Clin. Plast. Surg..

[13] Salibian A.A., Bekisz J.M., Frey J.D., Nolan I.T., Kaoutzanis C., Yu J.W., Levine J.P., Karp N.S., Choi M., Thanik V.D. (2021). Comparing Outcomes between Stacked/Conjoined and Non-Stacked/Conjoined Abdominal Microvascular Unilateral Breast Reconstruction. Microsurgery.

[14] Murray A., Wasiak J., Rozen W.M., Ferris S., Grinsell D. (2015). Stacked Abdominal Flap for Unilateral Breast Reconstruction. J. Reconstr. Microsurg..

[15] Rozen W.M., Patel N.G., Craggs B.S., Ramakrishnan V.V. (2016). Four-Flap Breast Reconstruction with Bilateral Stacked Flaps. Plast. Reconstr. Surg..

[16] Mayo J.L., Allen R.J., Sadeghi A. (2015). Four-Flap Breast Reconstruction: Bilateral Stacked DIEP and PAP Flaps. Plast. Reconstr. Surg. Glob. Open.

[17] Haddock N.T., Suszynski T.M., Teotia S.S. (2020). Consecutive Bilateral Breast Reconstruction Using Stacked Abdominally Based and Posterior Thigh Free Flaps. Plast. Reconstr. Surg..

[18] Haddock N.T., Cho M.J., Teotia S.S. (2019). Comparative Analysis of Single versus Stacked Free Flap Breast Reconstruction: A Single-Center Experience. Plast. Reconstr. Surg..

[19] Haddock N.T., Cho M.J., Gassman A., Teotia S.S. (2019). Stacked Profunda Artery Perforator Flap for Breast Reconstruction in Failed or Unavailable Deep Inferior Epigastric Perforator Flap. Plast. Reconstr. Surg..

[20] Dellacroce F.J., Sullivan S.K., Trahan C. (2011). Stacked Deep Inferior Epigastric Perforator Flap Breast Reconstruction: A Review of 110 Flaps in 55 Cases over 3 Years. Plast. Reconstr. Surg..

[21] Blondeel P.N., Hijjawi J., Depypere H., Roche N., Van Landuyt K. (2009). Shaping the Breast in Aesthetic and Reconstructive Breast Surgery: An Easy Three-Step Principle. Part II-Breast Reconstruction after Total Mastectomy. Plast. Reconstr. Surg..

[22] Chang C.S., Lanni M.A., Mirzabeigi M.N., Bucky L.P. (2022). Large-Volume Fat Grafting: Identifying Risk Factors for Fat Necrosis. Plast. Reconstr. Surg..

[23] Wederfoort J.L.M., Hebels S.A., Heuts E.M., van der Hulst R.R.W.J., Piatkowski A.A. (2022). Donor Site Complications and Satisfaction in Autologous Fat Grafting for Breast Reconstruction: A Systematic Review. J. Plast. Reconstr. Aesthetic Surg..

[24] Haddock N.T., Suszynski T.M., Teotia S.S. (2020). An Individualized Patient-Centric Approach and Evolution towards Total Autologous Free Flap Breast Reconstruction in an Academic Setting. Plast. Reconstr. Surg. Glob. Open.

[25] Haddock N.T., Teotia S.S. (2020). Consecutive 265 Profunda Artery Perforator Flaps: Refinements, Satisfaction, and Functional Outcomes. Plast. Reconstr. Surg. Glob. Open.

[26] Haddock N.T., Teotia S.S. (2020). Lumbar Artery Perforator Flap: Initial Experience with Simultaneous Bilateral Flaps for Breast Reconstruction. Plast. Reconstr. Surg. Glob. Open.

[27] Haddock N.T., Dumestre D.O., Teotia S.S. (2020). Efficiency in DIEP Flap Breast Reconstruction: The Real Benefit of Computed Tomographic Angiography Imaging. Plast. Reconstr. Surg..

[28] Teotia S.S., Dumestre D.O., Jayaraman A.P., Sanniec K.J., Haddock N.T. (2020). Revisiting Anastomosis to the Retrograde Internal Mammary System in Stacked Free Flap Breast Reconstruction: An Algorithmic Approach to Recipient-Site Selection. Plast. Reconstr. Surg..

[29] Koolen P.G.L., Lee B.T., Lin S.J., Erhard H.A., Greenspun D.T. (2015). Bipedicle-Conjoined Perforator Flaps in Breast Reconstruction. J. Surg. Res..

[30] Hamdi M., Khuthaila D.K., Van Landuyt K., Roche N., Monstrey S. (2007). Double-Pedicle Abdominal Perforator Free Flaps for Unilateral Breast Reconstruction: New Horizons in Microsurgical Tissue Transfer to the Breast. J. Plast. Reconstr. Aesthetic Surg..

[31] Cho M.J., Haddock N.T., Teotia S.S. (2020). Clinical Decision Making Using CTA in Conjoined, Bipedicled DIEP and SIEA for Unilateral Breast Reconstruction. J. Reconstr. Microsurg..

[32] Haddock N.T., Gassman A., Cho M.J., Teotia S.S. (2017). 101 Consecutive Profunda Artery Perforator Flaps in Breast Reconstruction: Lessons Learned with Our Early Experience. Plast. Reconstr. Surg..

[33] Cho M.J., Haddock N.T., Gassman A.A., Teotia S.S. (2018). Use of Composite Arterial and Venous Grafts in Microsurgical Breast Reconstruction: Technical Challenges and Lessons Learned. Plast. Reconstr. Surg..

[34] Haddock N.T., Kelling J.A., Teotia S.S. (2021). Simultaneous Circumferential Body Lift and Four-Flap Breast Reconstruction Using Deep Inferior Epigastric Perforator and Lumbar Artery Perforator Flaps. Plast. Reconstr. Surg..

[35] Mericli A.F., McHugh T., Kruse B., DeSnyder S.M., Rebello E., Offodile A.C. (2020). Time-Driven Activity-Based Costing to Model Cost Utility of Enhanced Recovery after Surgery Pathways in Microvascular Breast Reconstruction. J. Am. Coll. Surg..

[36] Haddock N.T., Garza R., Boyle C.E., Liu Y., Teotia S.S. (2021). Defining Enhanced Recovery Pathway with or without Liposomal Bupivacaine in Diep Flap Breast Reconstruction. Plast. Reconstr. Surg..

